# Supplementary material for: Ultra‐Low‐Field Paediatric MRI in Low‐ and Middle‐Income Countries: Super‐Resolution Using a Multi‐Orientation U‐Net
Source: Hum Brain Mapp. 2024 Dec 30;46(1):e70112. doi: 10.1002/hbm.70112 (PMC11685182; doi:10.1002/hbm.70112)
Supplement: Supplementary file 1 — Data S1 [file HBM-46-e70112-s001.docx]

**Supplementary Information for:**

**Ultra-low-field paediatric MRI in low- and middle-income countries: super-resolution using a multi-orientation U-Net**

Levente Baljer, Yiqi Zhang, Niall J Bourke, Kirsten A Donald, Layla E Bradford, Jessica E Ringshaw, Simone R Williams, Sean CL Deoni, Steven CR Williams, Khula SA Study Team, František Váša^*^, Rosalyn J Moran^*^


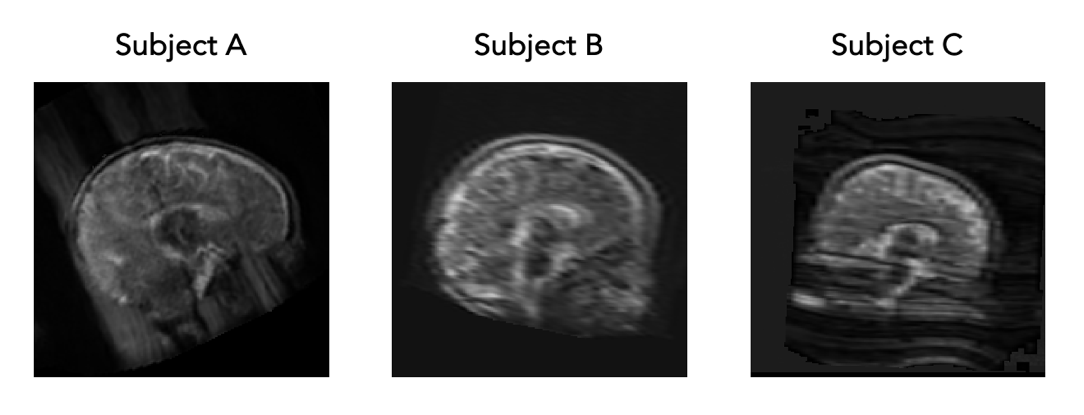


Figure S1) Sample of subjects who were excluded from the training set. All subjects A, B and C exhibit significant imaging artifacts that make them unsuitable for model training or inference.

Table S1) Demographic distribution across training, validation and test sets across all four folds.

|  | Age (months) | **Training set** | | **Validation set** | | **Test set** | |
| --- | --- | --- | --- | --- | --- | --- | --- |
|  |  | Male | Female | Male | Female | Male | Female |
| Fold 1 | 3 | 6 (14.3%) | 6 (14.3%) | 1 (14.3%) | 1 (14.3%) | 0 (0.0%) | 1 (14.3%) |
|  | 6 | 17 (40.5%) | 13 (30.9%) | 3 (42.8%) | 2 (28.6%) | 3 (42.8%) | 3 (42.8%) |
| Fold 2 | 3 | 5 (11.9%) | 5 (11.9%) | 1 (14.3%) | 2 (28.6%) | 1 (14.3%) | 1 (14.3%) |
|  | 6 | 19 (45.3%) | 13 (30.9%) | 2 (28.6%) | 2 (28.6%) | 2 (28.6%) | 3 (42.8%) |
| Fold 3 | 3 | 5 (11.9%) | 6 (14.3%) | 1 (14.3%) | 1 (14.3%) | 1 (14.3%) | 1 (14.3%) |
|  | 6 | 17 (40.5%) | 14 (33.3%) | 3 (42.8%) | 2 (28.6%) | 3 (42.8%) | 2 (28.6%) |
| Fold 4 | 3 | 5 (11.9%) | 6 (14.3%) | 1 (14.3%) | 1 (14.3%) | 1 (14.3%) | 1 (14.3%) |
|  | 6 | 16 (38.1%) | 15 (35.7%) | 4 (57.1%) | 1 (14.3%) | 3 (42.8%) | 2 (28.6%) |

Table S2) Image quality assessment of predictions from MO U-Nets trained on varying loss functions (L1, L2, L2 + LPIPS). All models were trained for 1000 epochs on a subset of the data used in the final experiment

Figure S2) Model outputs from MO U-Nets trained on varying loss functions. Left to right: axial ULF scan, MO U-Net output with L1 loss, L2 loss, L2 + LPIPS loss, ground-truth HF scan.

| **Loss function used** | **NMSE (↓)** | **PSNR (↑)** | **SSIM (↑)** |
| --- | --- | --- | --- |
| L1 loss | 0.0835 | 29.504 | 0.875 |
| L2 loss | 0.807 | 29.642 | 0.878 |
| L2 + LPIPS loss | **0.708** | **30.200** | **0.885** |


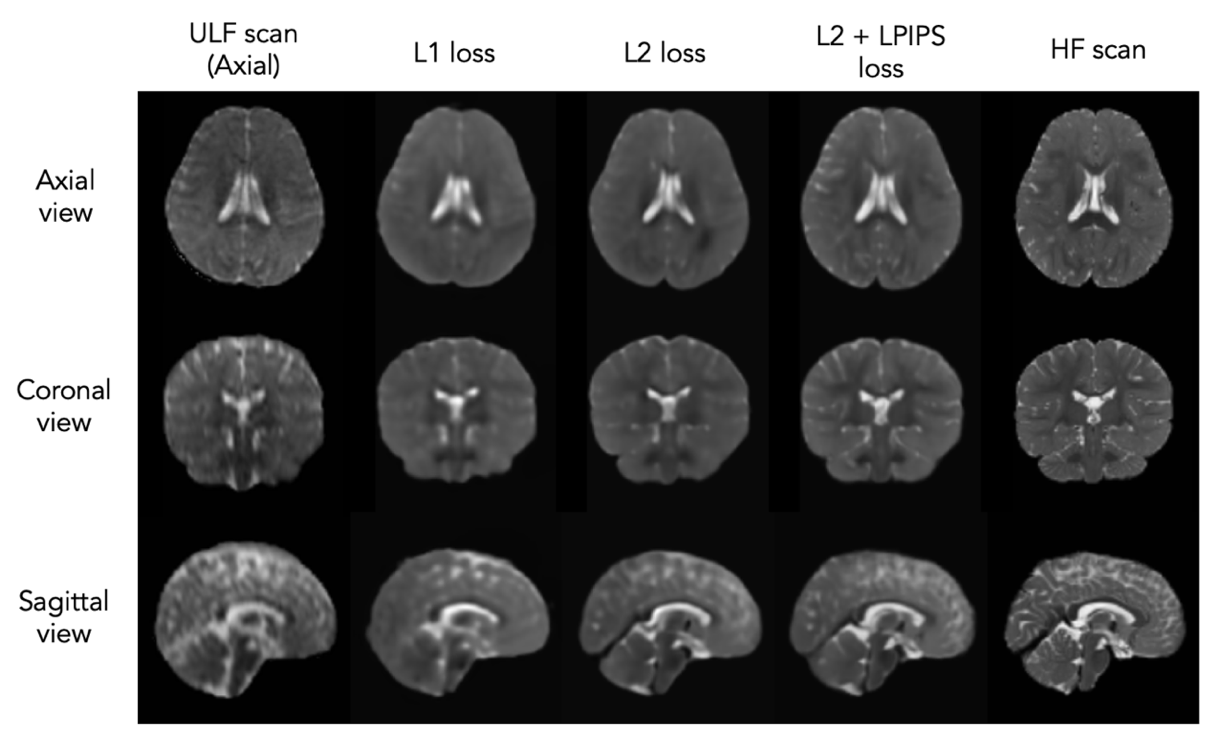


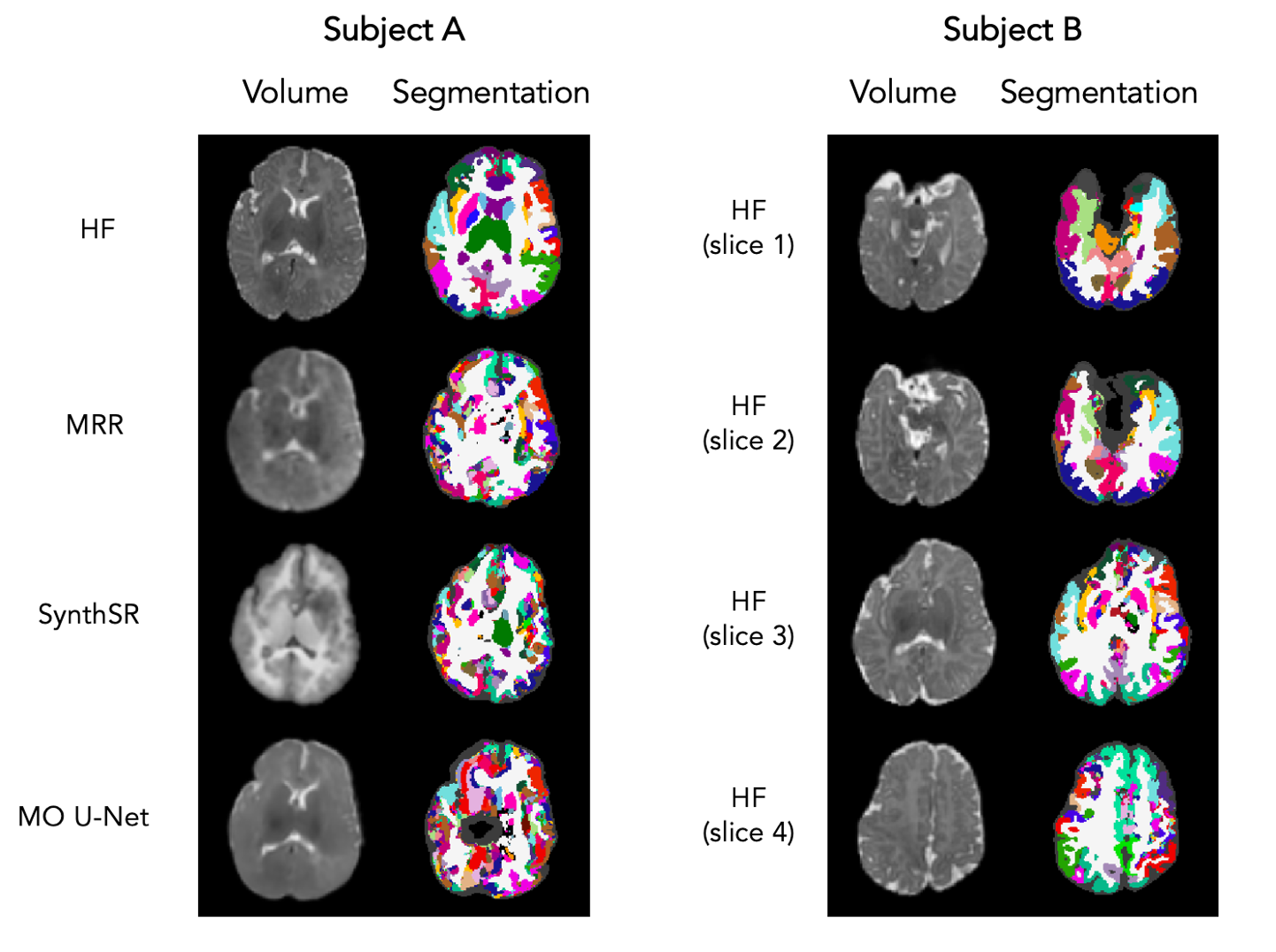


Figure S3) Test subjects excluded from segmentation-based analyses, owing to poor SynthSeg outputs. A) Subject with acceptable HF segmentation, but failed segmentations for all three SR outputs (MRR, SynthSR, and MO U-Net). B) Subject with failed HF segmentation, preventing comparison to ground-truth. Both subjects excluded had scans taken at 3-months of age.

Table S3) Distribution of sex and age across test set. Row 1: total test subjects; Row 2: test subjects for segmentation-based analyses. where two subjects with failed segmentations were excluded; Row 3: test subjects for image quality metrics (NMSE, PSNR, SSIM), where three subjects with no T_1_w HF scan were excluded

| **Number of subjects** | **Sex** | | **Age** | |
| --- | --- | --- | --- | --- |
|  | Male | Female | 3-months | 6-months |
| Total (N=28) | 14 | 14 | 7 | 21 |
| Segmentation (N=26) | 13 | 13 | 5 | 21 |
| Image quality (N=25) | 13 | 12 | 6 | 19 |

Table S4) Median Dice overlap with segmentations from HF scans, across global tissue types. Scores are stratified according to age group: 3-months (N=5) and 6-months (N=21). Both values are shown for ULF scans, MRR outputs, SynthSR outputs and MO U-Net outputs

| **Region** | **ULF (avg)** | | **MRR** | | **SynthSR** | | **MO U-Net** | |
| --- | --- | --- | --- | --- | --- | --- | --- | --- |
|  | 3M | 6M | 3M | 6M | 3M | 6M | 3M | 6M |
| GMC | 0.553 | 0.635 | 0.726 | 0.719 | 0.657 | 0.654 | 0.726 | 0.742 |
| GMS | 0.054 | 0.585 | 0.752 | 0.828 | 0.748 | 0.847 | 0.843 | 0.864 |
| WM | 0.469 | 0.633 | 0.734 | 0.760 | 0.718 | 0.753 | 0.708 | 0.776 |
| CSF | 0.208 | 0.332 | 0.530 | 0.509 | 0.427 | 0.408 | 0.520 | 0.548 |
| **Average** | 0.321 | **0.546** | 0.686 | **0.704** | 0.638 | **0.666** | 0.699 | **0.732** |


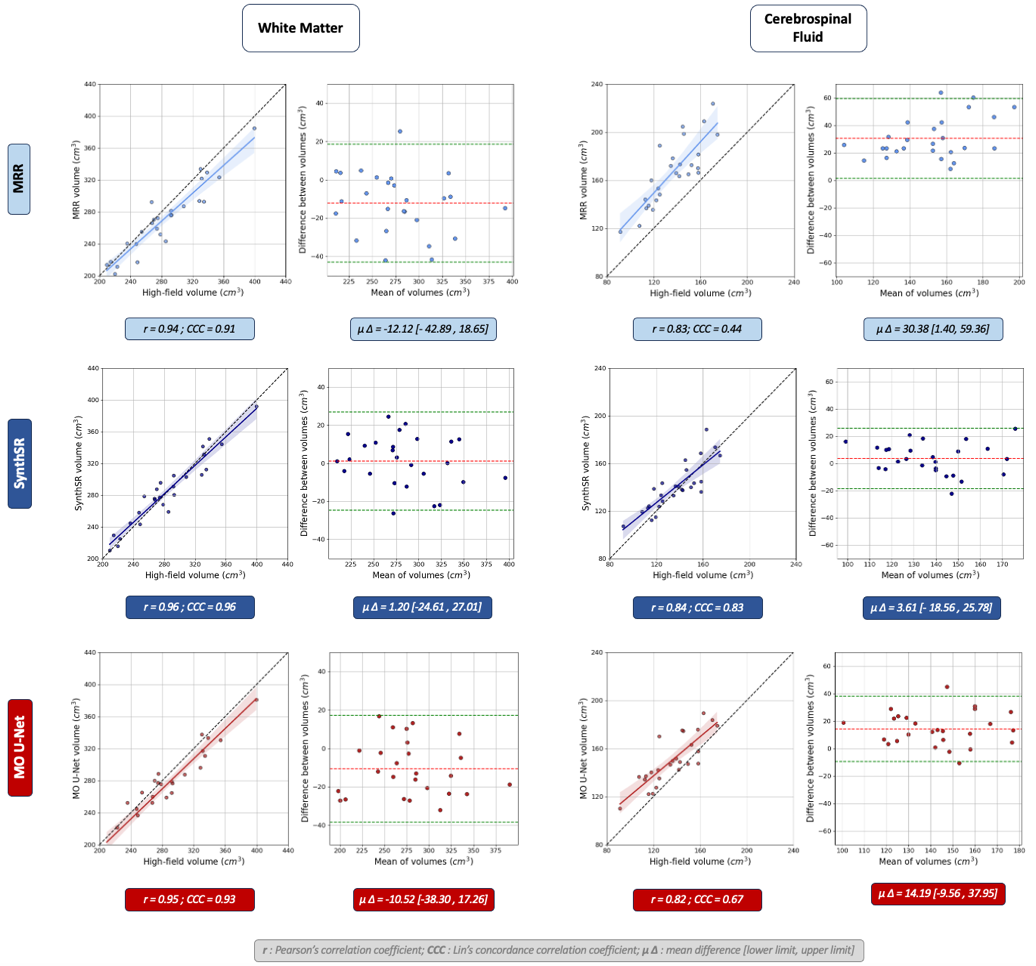

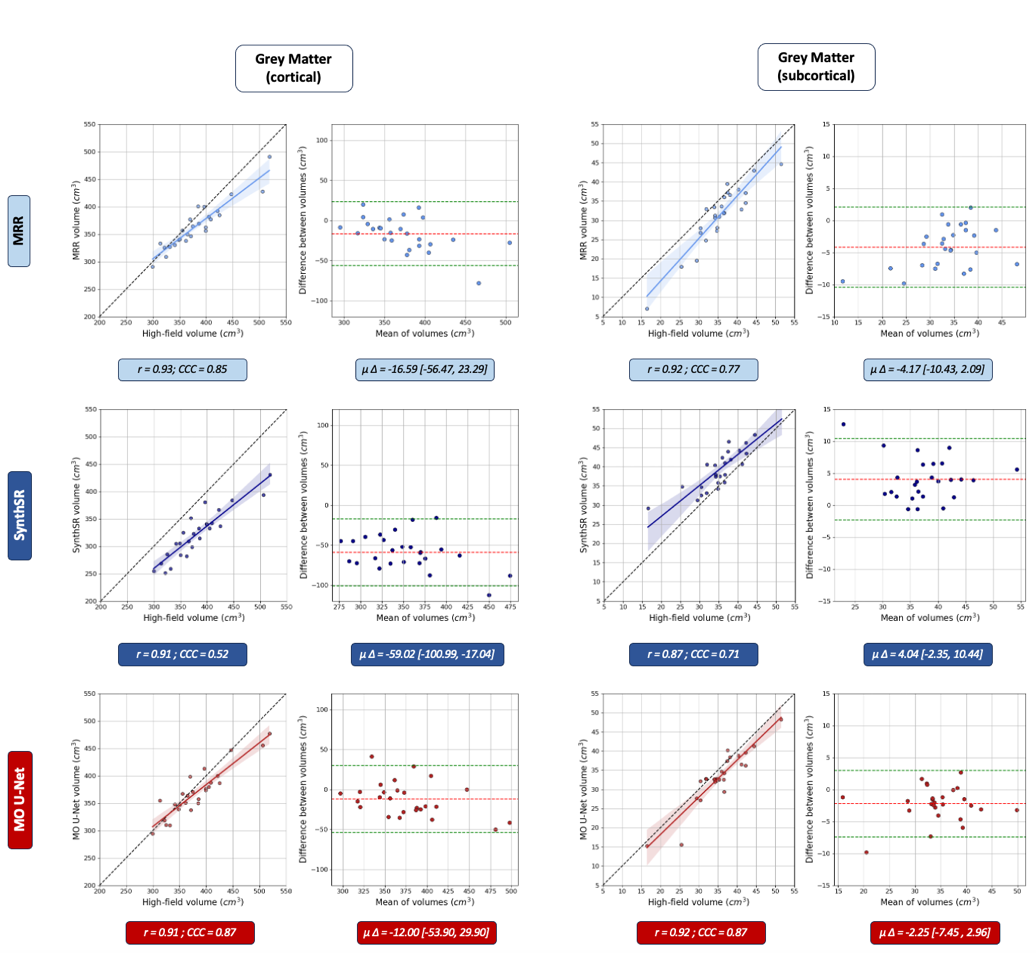


Figure S4) Tissue-type-specific volume analysis between MRR outputs and ground-truth HF scans (light blue), SynthSR outputs and ground-truth HF-scans (dark blue), and MO U-Net outputs and ground-truth HF scans (red). For each SR method and tissue type, we display volume correlation (left) and Bland-Altman plot (right). Tissue types, from left to right, top to bottom: cortical grey matter, subcortical grey matter, white matter, cerebrospinal fluid.

Table S5) Median Dice overlap with segmentations from HF scans, across subcortical regions. Columns 2-5: average of ULF scans (axial, coronal and sagittal), MRR outputs, SynthSR outputs, MO U-Net outputs.

| **Region** | **ULF (avg)** | **MRR** | **SynthSR** | **MO U-Net** |
| --- | --- | --- | --- | --- |
| Accumbens | 0.272 | 0.673 | 0.703 | 0.715 |
| Amygdala | 0.156 | 0.720 | 0.782 | 0.766 |
| Caudate | 0.412 | 0.747 | 0.735 | 0.803 |
| Hippocampus | 0.256 | 0.698 | 0.708 | 0.764 |
| Pallidum | 0.289 | 0.625 | 0.799 | 0.741 |
| Putamen | 0.456 | 0.781 | 0.819 | 0.838 |
| Thalamus | 0.579 | 0.872 | 0.870 | 0.906 |
| Ventral DC | 0.374 | 0.786 | 0.808 | 0.798 |
| **Average** | 0.349 | 0.738 | 0.778 | **0.791** |

Table S6) Wilcoxon signed-rank test applied to Dice scores of 26 test subjects, comparing outputs of MO U-Net to MRR, and MO U-Net to SynthSR. For each comparison, the rank biserial correlation (RBC) and associated significance of the underlying Wilcoxon signed-rank test are displayed. Analysis is centred on subcortical regions. FWR = 0.003125

| **Region** | **MO U-Net > MRR** | | **MO U-Net > SynthSR** | |
| --- | --- | --- | --- | --- |
|  | *RBC* | *Sig.* | *RBC* | *Sig.* |
| Accumbens | 0.385 | 0.045 | 0.569 | 0.57 |
| Amygdala | 0.556 | 0.0060 | 0.037 | 0.44 |
| Caudate | 0.749 | 0.00021^*^ | 0.442 | 0.025 |
| Hippocampus | 0.943 | <0.0001^*^ | 0.601 | 0.0031 |
| Pallidum | 0.778 | 0.00011^*^ | -0.413 | 0.97 |
| Putamen | 0.875 | <0.0001^*^ | 0.162 | 0.24 |
| Thalamus | 0.726 | 0.00033^*^ | 0.675 | 0.00089^*^ |
| Ventral DC | 0.527 | 0.0088 | 0.265 | 0.12 |

Table S7) Median Dice overlap with segmentations from HF scans, across subcortical regions. Scores are stratified according to age group: 3-months (N=5) and 6-months (N=21). Both values are shown for ULF scans, MRR outputs, SynthSR outputs and MO U-Net outputs

| **Region** | **ULF (avg)** | | **MRR** | | **SynthSR** | | **MO U-Net** | |
| --- | --- | --- | --- | --- | --- | --- | --- | --- |
|  | 3M | 6M | 3M | 6M | 3M | 6M | 3M | 6M |
| Accumbens | 0.010 | 0.313 | 0.518 | 0.694 | 0.667 | 0.716 | 0.648 | 0.723 |
| Amygdala | 0.0 | 0.301 | 0.441 | 0.721 | 0.671 | 0.783 | 0.664 | 0.774 |
| Caudate | 0.006 | 0.445 | 0.679 | 0.771 | 0.646 | 0.764 | 0.781 | 0.804 |
| Hippocampus | 0.0 | 0.320 | 0.385 | 0.703 | 0.512 | 0.729 | 0.559 | 0.778 |
| Pallidum | 0.0 | 0.308 | 0.448 | 0.630 | 0.534 | 0.804 | 0.675 | 0.754 |
| Putamen | 0.010 | 0.515 | 0.690 | 0.781 | 0.735 | 0.833 | 0.804 | 0.841 |
| Thalamus | 0.0 | 0.654 | 0.806 | 0.875 | 0.806 | 0.876 | 0.904 | 0.910 |
| Ventral DC | 0.0 | 0.432 | 0.701 | 0.793 | 0.711 | 0.815 | 0.781 | 0.800 |
| **Average** | 0.003 | **0.411** | 0.584 | **0.746** | 0.660 | **0.790** | 0.727 | **0.798** |

Table S8) Pearson’s r, Lin’s CCC, and mean estimated volume difference as calculated for each SR technique. MRR = multi-resolution registration, SSR = SynthSR, MOU = multi-orientation U-Net

| **Region** | **Pearson’s r** | | | **Lin’s CCC** | | | **Volume μ Δ (cm^3^)** | | |
| --- | --- | --- | --- | --- | --- | --- | --- | --- | --- |
|  | MRR | SSR | MOU | MRR | SSR | MOU | MRR | SSR | MOU |
| Accumbens | 0.627 | 0.583 | 0.544 | 0.599 | 0.336 | 0.524 | 0.057 | 0.220 | 0.003 |
| Amygdala | 0.800 | 0.858 | 0.827 | 0.683 | 0.570 | 0.791 | -0.238 | 0.439 | -0.127 |
| Caudate | 0.839 | 0.775 | 0.935 | 0.800 | 0.490 | 0.934 | -0.359 | 1.016 | -0.047 |
| Hippocampus | 0.835 | 0.822 | 0.861 | 0.638 | 0.408 | 0.860 | -0.899 | 1.356 | -0.056 |
| Pallidum | 0.806 | 0.784 | 0.807 | 0.486 | 0.712 | 0.594 | -0.598 | 0.245 | -0.439 |
| Putamen | 0.798 | 0.734 | 0.829 | 0.529 | 0.704 | 0.583 | -1.193 | -0.127 | -1.004 |
| Thalamus | 0.916 | 0.714 | 0.918 | 0.839 | 0.649 | 0.901 | -0.721 | 0.350 | -0.246 |
| Ventral DC | 0.840 | 0.790 | 0.770 | 0.705 | 0.649 | 0.688 | -0.219 | 0.546 | -0.332 |
| **Average** | 0.808 | 0.758 | **0.811** | 0.660 | 0.537 | **0.734** | -0.521 | 0.506 | **-0.281** |

Table S9) Image quality metrics (NMSE, PSNR, SSIM) for SR methods generating T_2_w scans: MRR and MO U-Net. Values are obtained by comparing SR outputs with ground-truth HF scans. The analyses are conducted on all N=28 test subjects.

| **SR Method** | **NMSE (↓)** | **PSNR (↑)** | **SSIM (↑)** |
| --- | --- | --- | --- |
| MRR | 0.166 | 26.164 | 0.447 |
| MO U-Net | **0.068** | **30.527** | **0.901** |

Table S10) Image quality metrics (NMSE, PSNR, SSIM) for each SR method: MRR, SynthSR and MO U-Net. Scores are stratified according to age group: 3-months (N=6) and 6-months (N=19)

| **SR**  **Method** | **NMSE (↓)** | | **PSNR (↑)** | | **SSIM (↑)** | |
| --- | --- | --- | --- | --- | --- | --- |
|  | 3-month | 6-month | 3-month | 6-month | 3-month | 6-month |
| MRR | **0.126** | 0.175 | **27.770** | 26.357 | **0.494** | 0.438 |
| SynthSR | **0.277** | 0.954 | **24.032** | 20.539 | **0.878** | 0.870 |
| MO U-Net | **0.063** | **0.063** | **30.826** | 30.810 | 0.875 | **0.915** |

Table S11) Dice scores between HF segmentations and segmentations obtained from MO U-Net outputs, across subcortical regions. MO U-Net scores are further stratified according to how many unique inputs the model received (A = axial, A/S = axial + sagittal, A/C/S = axial + sagittal + coronal). For comparison with ULF scans, MRR outputs and SynthSR outputs, see Table S5.

| **Region** | **MO U-Net** | | |
| --- | --- | --- | --- |
|  | Axial | Axial/Sagittal | Axial/Coronal/Sagittal |
| Accumbens | 0.707 | 0.717 | 0.715 |
| Amygdala | 0.724 | 0.765 | 0.766 |
| Caudate | 0.813 | 0.808 | 0.803 |
| Hippocampus | 0.738 | 0.744 | 0.764 |
| Pallidum | 0.713 | 0.728 | 0.741 |
| Putamen | 0.818 | 0.829 | 0.838 |
| Thalamus | 0.898 | 0.896 | 0.906 |
| Ventral DC | 0.785 | 0.801 | 0.798 |
| **Average** | 0.775 | 0.786 | **0.791** |

Table S12) Portion of total subjects having completed three separate T_2_w ULF scans (axial, coronal and sagittal), compared to those who additionally completed a T_1_w axial scan.

| **Total subjects** | **T_2_w Axial/Coronal/Sagittal** | **T_1_w Axial** |
| --- | --- | --- |
| 82 | 63 | 35 |

**Khula SA Study Team**

Michal R. Zieff

Donna Herr

Chloë A. Jacobs

Sadeeka Williams

Zamazimba Madi

Nwabisa Mlandu

Tembeka Mhlakwaphalwa

Lauren Davel

Reese Samuels

Zayaan Goolam

Thandeka Mazubane

Bokang Methola

Khanyisa Nkubungu

Candice Knipe
